# Human islet xenotransplantation in rodents: A literature review of experimental model trends

**DOI:** 10.6061/clinics/2017(04)08

**Published:** 2017-04

**Authors:** Leandro Ryuchi Iuamoto, André Silva Franco, Fábio Yuji Suguita, Felipe Futema Essu, Lucas Torres Oliveira, Juliana Mika Kato, Matheus Belloni Torsani, Alberto Meyer, Wellington Andraus, Eleazar Chaib, Luiz Augusto Carneiro D'Albuquerque

**Affiliations:** I Faculdade de Medicina, Universidade de Sao Paulo, Sao Paulo, SP, BR; II Departamento de Gastroenterologia, Faculdade de Medicina, Universidade de Sao Paulo, Sao Paulo, SP, BR

**Keywords:** Islet Transplantation, Allograft, Transplantation, Heterologous, Islets of Langerhans

## Abstract

Among the innovations for the treatment of type 1 diabetes, islet transplantation is a less invasive method of treatment, although it is still in development. One of the greatest barriers to this technique is the low number of pancreas donors and the low number of pancreases that are available for transplantation. Rodent models have been chosen in most studies of islet rejection and type 1 diabetes prevention to evaluate the quality and function of isolated human islets and to identify alternative solutions to the problem of islet scarcity. The purpose of this study is to conduct a review of islet xenotransplantation experiments from humans to rodents, to organize and analyze the parameters of these experiments, to describe trends in experimental modeling and to assess the viability of this procedure. In this study, we reviewed recently published research regarding islet xenotransplantation from humans to rodents, and we summarized the findings and organized the relevant data. The included studies were recent reports that involved xenotransplantation using human islets in a rodent model. We excluded the studies that related to isotransplantation, autotransplantation and allotransplantation. A total of 34 studies that related to xenotransplantation were selected for review based on their relevance and current data. Advances in the use of different graft sites may overcome autoimmunity and rejection after transplantation, which may solve the problem of the scarcity of islet donors in patients with type 1 diabetes.

## INTRODUCTION

According to the International Diabetes Federation (IDF), diabetes mellitus currently affects 382 million people, with a projected increase to 592 million people by 2035 1.

The etiology of type I diabetes mellitus is unknown; however, histopathological findings indicate an autoimmune destruction of ß-cells, an association with HLA alleles and environmental factors, such as exposure to bovine milk. Diabetes mellitus was historically considered a fatal disease that resulted in hyperglycemic coma. However, since the discovery of the therapeutic application of insulin in the 1920s, diabetes mellitus has become a chronic disease that causes many complications, including retinopathy, nephropathy, vasculopathy and neuropathy.

In 1894, the first case of islet transplantation as a treatment for diabetes was described by Dr. Watson Williams and Hareshant. Notably, this case occurred before the insulin isolation of Banting, Best and Collip in 1921. In the early twentieth century, Dr. W. Williams attempted to implant sheep pancreatic fragments in the subcutaneous tissue of a 15-year-old male with ketoacidosis. However, the xenograft was rejected because of a lack of immunosuppressive techniques. In 1972, Dr. P. Lacey demonstrated the reversibility of diabetes in rodents by using islet implantation 2.

The first successes in islet allografts in the surgical treatment of diabetes occurred in 1990 with Scharp et al., who achieved insulin independence in a patient with type 1 diabetes mellitus for one month. However, many technical difficulties were found during the reproduction of this experiment.

One of the greatest barriers to the development of islet transplantation is the low number of pancreas donors and the low number of pancreases that can be used for transplantation 3. According to the Network of Organ Procurement and Transplantation, fewer than 20% of the pancreases that are collected from a total of 8,000 donors are available for transplantation. In addition, many pancreas donors do not meet the selection criteria, and many islets are handled incorrectly, negatively affecting the transplant procedure. 4 Other inconveniences are the high cost of islet isolation, the poor durability of insulin independence, autoimmunity and rejection after transplantation 2,3,5,6.

To supply the scarcity of islets, animal donors, such as pigs, could provide an alternative source of cells for transplantation 7. However, xenotransplantation is challenged by the possible risk of infection from pathogens within the donor animal. Specifically, all pigs contain multiple copies of porcine endogenous retrovirus and at least three variants of pig endogenous retrovirus (PERV), which can infect human cells in vitro. Thus, there is a risk of PERV infection associated with the xenotransplantation of pig islets to immunosuppressed human patients 8,9.

In this context, to evaluate the quality and function of isolated human islets 10, the rodent has been chosen over other animals in most studies that involve islet rejection and the prevention of type 1 diabetes 3.

Manikandan et al. 11 studied the antioxidant effect of black tea on the regeneration of pancreatic ß-cells and observed a positive therapeutic effect in rodent studies. Recently, Gu et al. 12 described an alternative therapeutic strategy to treat type 1 diabetes, namely, treatment by nanoparticles, which sustainably promotes the self-regulation of glucose-mediated insulin secretion. This effect is observed for a longer period of time than the insulin injections that are currently used for treatment.

Although there have been many positive results related to the xenotransplantation of human islets to rodents, researchers have rarely achieved a breakthrough in the clinical treatment of islet transplantation, perhaps because of the differences between the human immune system and the rodent model. These differences have stimulated the development of humanized rodent models, which allow the detailed study of human immune system cells and transplanted human islets in vivo 3.

The purpose of this study is to review islet xenotransplantation experimental attempts from humans to rodents, to organize the parameters of these experiments and to analyze the viability of these procedures.

## METHODOLOGY

We reviewed studies regarding islet xenotransplantation from humans to rodents. The relevant data from recently published studies from 2006 to 2016 were summarized and organized.

### Eligibility Criteria

#### Types of Studies

The study designs of previous reviews and experimental studies were included.

#### Types of Participants

Donor participants were humans from whom islets were isolated and transplanted to rodents (recipient).

#### Types of Intervention

The interventions were islet xenotransplantation from humans to rodents. There were different graft sites and types of islet recipients. In the present review, only the studies that relate to human to rodent islet xenotransplantation were selected.

#### Types of Parameters Analyzed

Several parameters were considered, namely, strain, gender, age and weight of the recipient, xenotransplantation site, graft survival time (follow up), number of transplanted islets and diabetes induction method.

### Exclusion Criteria

Articles discussing transplantation in porcine, tilapia and nonhuman primates (which are some of the more common species that are used for transplantation) were excluded from the review to focus on the articles that relate to islet xenotransplantation from humans to rodents. Studies using stem cells or that had an unclear methodology were excluded from our review.

Research letters, articles not published in English and articles for which the full text was unavailable were not considered in this review.

Following the PubMed search, we reviewed the references from the retrieved publications and obtained the entire text of the publications for potential inclusion in the review.

### Literature Search

Using the Medline database, the literature was searched for English-language articles that were published from January 2006 to January 2016.

We performed a manual search of the references and contacted experts in the field.

#### Search Strategy

We searched for published articles by using the Medline database with the keywords "rodent islet transplantation".

We also selected the most recent works that were published from January 2006 to January 2016 by using the following search terms: “(((((rodent human islet xenotransplantation) NOT tilapia) NOT porcine) NOT nonhuman primate) NOT pig) AND (“2006”[Date - Completion]: “2016”[Date - Completion])”.

Articles that were published before 2006 were not included in the analysis because of a lack of information, relevance and current data.

#### Data Extraction

The data from each study were independently extracted by 3 of the authors. Disagreements were resolved by consensus. If no consensus was achieved, a fourth author was consulted.

## RESULTS

A total of 1,819 articles from 2006 to 2016 were found, but only 225 articles were related to xenotransplantation and were thus selected based on their relevance and current information. We selected 91 articles and analyzed them; 34 of these articles were had good methodological quality, such as updated information that is necessary for this review and a description of all comparative parameters related to islet xenotransplantation from human donors to rodents.

According to the selected studies, C57BL/6 mice were the most used strains in xenograft experiments as islet recipients (22%), followed by NOD-SCID and BALB/c mice (14% each), SCID mice (8%), and NU/NU mice (6%). Syrian Golden hamsters, athymic nude Foxn1-nu mice, NOD/LtJ mice, NOD SCID gamma mice, Rowett rats, and SCID-Beige mice were the least commonly used recipients (3% each).

The results are organized and displayed in Tables 1 and 2.

## DISCUSSION

Islet transplantation is an innovation for type 1 diabetes treatment that is less invasive and that has a 20-fold lower morbidity rate than pancreas transplantation 2,4,6,16.

Some studies have reported an 80% rate of insulin independence during the first postoperative year in the patients who were treated with islet transplantation. However, graft survival rates remain low 2.

The islet transplantation technique has been developed to provide an adequate supply of insulin, which solves the problem of donor shortage for diabetic patients 17. From 1991 to 2000, 450 islet transplantation attempts were performed in patients with type 1 diabetes with only an 8% success rate.

We discuss the analyzed studies in more detail below.

### Recipient characteristics

In this study, we reviewed the articles describing xenograft transplantation in rodents. The majority of the animals were between 9 and 16 weeks old and were male (32.4% male; 17.6% female; 50% N/A). See Table 1. Although more studies used C57BL/6 mice in the xenograft experiments (22%), followed by NOD-SCID and BALB/c mice (14% each), no significant difference was observed in the results that were obtained using other strains.

### Diabetes induction method

The standard diabetes induction method was the use of streptozotocin. The median dose was 170 mg/kg (50-250 mg/kg).

### Islet xenotransplantation site

The authors used different sites for the xenografts (Table 2), but the kidney capsule (91.2% of the studies) was the most frequently used site for transplantation. Other sites, such as the intraperitoneal space, liver (portal vein), subcutaneous space, submandibular gland and dorsal window model, were used in a small number of studies.

The highest graft survival time was more than 300 days, which was obtained by Brehm MA et al. 19. This study used the subrenal space as the site of xenograft transplantation. Other studies that used the kidney capsule as the xenotransplantation site, such as the studies by Zhang J et al. 20, Sklavos MM et al. 21, Yamamoto T et al. 22 Scharfmann R et al. 23, Pearson T et al. 24, Vlad G et al. 25 and Fornoni A et al. 26, reported more than 100 days of graft survival time. Although the majority of articles show higher survival rates using sites that involve the kidney, Qi M et al. 27 used an intraperitoneal site and obtained 134 days (±17) of graft survival. Few articles have explored different xenograft sites, and it may thus be difficult to conclude whether these locations provide better graft survival rates than the kidney.

It is important to note that in many studies, the recipients were sacrificed for histopathological analysis.

We identified many variables on the analyzed studies. The characteristics of the xenotransplantation site are factors that can possibly influence the obtained results. Based on our analysis, it is possible to reproduce some of these studies and to modify additional variables to obtain better graft survival times. Nevertheless, one relevant limitation is that many studies did not describe the data that are essential to reproduce the described experiments, such as the strain, age and gender of the recipient animal and the diabetes induction method.

Although immunosuppressive drugs may increase the survival rates of islet allotransplantation in rodents by reducing the side effects 17, few studies have used immunosuppressants. It was therefore not possible to perform an analysis of the immunosuppressive effect in islet xenotransplantation. Future studies with improved methodologies are necessary to improve the graft survival time and to advance type 1 diabetes treatment.

The viability of pancreatic islet transplantation could be determined in only a small number of studies because of a lack of the information that is necessary to perform this procedure.

The survival rates in allograft experiments have increased with the use of novel graft sites. Different methodologies to conserve islets may overcome autoimmunity and rejection after transplantation and solve the problem of the scarcity of islet donors for patients with type 1 diabetes.

## AUTHOR CONTRIBUTIONS

Iuamoto LR, Franco AS, Suguita FY, Essu FF, Oliveira LT, Kato JM and Torsani MB were responsible for the literature review and manuscript writing. Iuamoto LR, Franco AS, Meyer A, Andraus W and D'Albuquerque LA were responsible for critical analysis. Iuamoto LR, Franco AS, Kato JM, Meyer A, Chaib E and D'Albuquerque LA were responsible for paper revision. Iuamoto LR, Franco AS, Meyer A, Chaib E, Andraus W and D'Albuquerque LA were responsible for manuscript review. Iuamoto LR and Meyer A were responsible for study design. Meyer A, Chaib E, Andraus W and D'Albuquerque LA were responsible for supervision of the study.

## Figures and Tables

**Table 1:** Comparative analysis of the types of rodents used and their clinical characteristics to evaluate the viability of the procedure: Strain, Gender, Age and Diabetes induction method.

Authors	Recipient	Gender	Age	Diabetes induction method	Viability	
					Yes	No
**Oh E, et al. 2014 28**	NOD-SCID mice	N/A	10-14 weeks	Streptozotocin 180 mg/kg	X	
**Wu DC, et al. 2013 14**	BALB/c mice	N/A	6-12 weeks	Streptozotocin 250 mg/kg	X	
**Brandhorst D, et al. 2013 29**	C57BL/6 mice	N/A	N/A	N/A		X
**Liu S, et al. 2013 30**	C57BL/6 mice	Male	10 weeks	Streptozotocin 200 mg/kg	X	
**Qi M, et al. 2012 27**	BALB/c mice	N/A	N/A	N/A		X
**Avgoustiniatos ES, et al. 2012 31**	N/A	N/A	N/A	Streptozotocin (dose: N/A)		X
**Noguchi H, et al. 2012 4**	N/A	N/A	N/A	Streptozotocin 220 mg/kg		X
**Pour PM, et al. 2012 32**	Syrian Golden hamsters	Female	8 years	Streptozotocin 50 mg/kg	X	
**McCall M, et al. 2011 33**	C57BL/6 mice	N/A	N/A	Streptozotocin (220mg/kg - BALB/c; 180mg/kg - B6-RAG-/-)		X
**Mwangi SM, et al. 2011 34**	athymic nude Foxn1-nu mice	N/A	6 weeks	Streptozotocin 75 mg/kg	X	
**Zhang J, et al. 2010 20**	NOD/LtJ mice	Female	N/A	N/A		X
**Sabek O, et al. 2010 35**	N/A	Female	10-12 weeks	N/A		X
**Rink JS, et al. 2010 36**	N/A	N/A	N/A	Streptozotocin 220 mg/kg		x
**Brehm MA, et al. 2010 19**	NOD SCID gamma mice	N/A	12-16 weeks	Spontaneous: 3-5 week-old	x	
**Sklavos MM, et al. 2010 21**	C57BL/6 and BALB/c	Male	6-8 weeks	Streptozotocin 240 mg/kg	x	
**Jacobs-Tulleneers-Thevissen D, et al. 2010 37**	Rowett rats	Male	7-10 weeks	Streptozotocin 60 mg/kg	x	
**Yamamoto T, et al. 2010 22**	N/A	N/A	N/A	Streptozotocin 200 mg/kg		x
**Toso C, et al. 2010 38**	C57BL/6 mice	Female and Male	N/A	Streptozotocin 175 mg/kg	x	
**Höglund E, et al. 2009 39**	C57BL/6 mice	Male	N/A	N/A		x
**Lee SH, et al. 2009 40**	SCID-Beige mice	N/A	8 weeks	Streptozotocin 40 mg/kg	x	
**Scharfmann R, et al. 2008 23**	SCID mice	Male	N/A	N/A		x
**Navarro-Alvarez N, et al. 2008 41**	SCID mice	Male	10-12 weeks	Streptozotocin 200 mg/kg	x	
**Pearson T, et al. 2008 24**	NOD-SCID mice	N/A	N/A	Streptozotocin 150 mg/kg		x
**Vlad G, et al. 2008 25**	NOD-SCID mice	Female	6-10 weeks	Streptozotocin 180 mg/kg	x	
**Papas KK, et al. 2007 42**	N/A	N/A	N/A	Streptozotocin (dose: N/A)		x
**Fornoni A, et al. 2007 26**	NU/NU mice	N/A	N/A	Streptozotocin 200 mg/kg		x
**Biancone L, et al. 2007 43**	BALB/c mice	Female	6-8 weeks	N/A		x
**Gao R, et al. 2006 44**	BALB/c mice	Male	6-8 weeks	N/A		x
**Cantaluppi V, et al. 2006 45**	SCID and C57BI/6 mice	N/A	N/A	N/A		x
**Sabek OM, et al. 2006 46**	NOD-SCID mice	N/A	N/A	Glucose 2 g/kg		x
**Lu Y, et al. 2006 47**	NOD-SCID mice	Male	8-12 weeks	streptozotocin 160 mg/kg	x	
**Fraker C, et al. 2006 48**	NU/NU mice	Male	N/A	Streptozotocin 200 mg/kg	x	
**Paulsson JF, et al. 2006 49**	N/A	Male	N/A	N/A		x
**Päth G, et al. 2006 50**	C57BL/6 mice	N/A	8-10 weeks	Streptozotocin (dose: N/A)	x	

**Table 2:** Preferred islet xenotransplantation site, number of transplanted islets and graft survival time (follow up).

Authors	Xenotransplantation site	Number of Transplanted Islets	Graft Survival Time(Follow up)
**Oh E, et al. 2014** 28	kidney capsule	100	15 days
**Wu DC, et al. 2013** 14	kidney subcapsular space	8,000	60 days
**Brandhorst D, et al. 2013** 29	kidney capsule	N/A	32 days
**Liu S, et al. 2013** 30	kidney capsule	200	over 90 days
**Qi M, et al. 2012** 27	intraperitoneal	N/A	151 days
**Avgoustiniatos ES, et al. 2012** 31	kidney capsule	1,000-2,000	N/A
**Noguchi H, et al. 2012** 4	kidney subcapsular space	1,200	30 days
**Pour PM, et al. 2012** 32	submandibular gland	750	84 days
**McCall M, et al. 2011** 33	kidney capsule	1,500	28 days
**Mwangi SM, et al. 2011** 34	kidney capsule	2,000	65 days
**Zhang J, et al. 2010** 20	kidney capsule	1,000	120 days
**Sabek O, et al. 2010** 35	dorsal window model	100	17 days
**Rink JS, et al. 2010** 36	kidney capsule	2,000	40 days
**Brehm MA, et al. 2010** 19	subrenal	4,000	over 300 days
**Sklavos MM, et al. 2010** 21	kidney capsule	100 or 175	over 120 days
**Jacobs-Tulleneers-Thevissen D, et al. 2010** 37	Liver - Portal vein; omental implants	N/A	N/A
**Yamamoto T, et al. 2010** 22	kidney capsule	1,000	120 days
**Toso C, et al. 2010** 38	kidney capsule	1,500	60 days
**Höglund E, et al. 2009** 39	kidney capsule	N/A	28 days
**Lee SH, et al. 2009** 40	renal subcapsular space	70	N/A
**Scharfmann R, et al. 2008** 23	kidney capsule	N/A	135 days
**Navarro-Alvarez N, et al. 2008** 41	subrenal kidney capsule	200	14 days
**Pearson T, et al. 2008** 24	renal subcapsular space	1,000-4,000	100 days
**Vlad G, et al. 2008** 25	kidney capsule	1,500	91 days
**Papas KK, et al. 2007** 42	kidney capsule	N/A	42 days
**Fornoni A, et al. 2007** 26	kidney subcapsular space	2000, 1,000 or 500	127 days
**Biancone L, et al. 2007** 43	kidney capsule	1,000	65 days
**Gao R, et al. 2006** 44	kidney capsule	5uL	90 days
**Cantaluppi V, et al. 2006** 45	subcutaneous	N/A	14 days
**Sabek OM, et al. 2006** 46	kidney capsule	2,000	14 days
**Lu Y, et al. 2006** 47	kidney capsule	1,500 and 2,500	30 days
**Fraker C, et al. 2006** 48	kidney capsule	2,000	60 days
**Paulsson JF, et al. 2006** 49	kidney capsule	N/A	28 days
**Päth G, et al., 2006** 50	kidney capsule	500	9 days
